# Pharmacological Activity of Kaurenoic Acid Nanocarriers and Formulation Considerations for Therapeutic Cancer Applications

**DOI:** 10.3390/pharmaceutics18040437

**Published:** 2026-04-01

**Authors:** Peter Ikechukwu, Remigius Agu

**Affiliations:** 1 Department of Pharmacology and Toxicology, University of Nigeria, Nsukka 410001, Enugu State, Nigeria; 2 Biopharmaceutics and Drug Delivery Lab, College of Pharmacy, Faculty of Health, Dalhousie University, P.O. Box 15000, Halifax, NS B3H 4R2, Canada

**Keywords:** kaurenoic acid, ent-kaurane diterpenoid, liposomes, nanocarriers, lipid-based drug delivery, pharmacokinetics, nanomedicine, nanocarrier formulation, bioavailability enhancement

## Abstract

Kaurenoic acid (KA) is an ent-kaurane diterpenoid present in several medicinal plant species and has been reported to exhibit anti-inflammatory, cytotoxic, and analgesic activity in experimental models. Despite its pharmacological profile, the development of KA as a therapeutic agent has been hindered by its unfavorable physicochemical and biopharmaceutical properties. KA is highly lipophilic and poorly soluble in water, which limits its dissolution, systemic exposure, and oral bioavailability. These limitations are common among plant-derived bioactive compounds and pose significant challenges for clinical development. Lipid-based nanocarrier systems, particularly liposomal formulations, have therefore been investigated as potential delivery strategies for improving the biopharmaceutical performance of KA. Encapsulating KA within phospholipid bilayers can improve its apparent solubility, protect it from degradation, and modify its biodistribution compared to the free compound. In this review, we discuss the pharmacological mechanisms of KA, its physicochemical properties, and the biopharmaceutical barriers to its therapeutic development. We also critically evaluate published studies on nanocarrier-based formulations, focusing on encapsulation efficiency, particle size, release properties, and pharmacokinetics (PK). Additionally, regulatory and pharmaceutical considerations relevant to lipid-based delivery of KA are addressed. Available evidence supports lipid-based nanocarriers as a promising strategy to improve preclinical development and formulation performance of poorly soluble plant bioactives such as kaurenoic acid. Although KA-loaded nanocarriers demonstrate encouraging activity in preclinical models, comprehensive pharmacokinetic and safety evaluations remain necessary before clinical development can be realistically considered.

## 1. Introduction

Kaurenoic acid (KA) is a naturally occurring ent-kaurane diterpenoid found in various medicinal plant species and studied for its pharmacological effects in models of inflammation, cancer, and pain (Table 1) [1,2,3,4]. The table summarizes botanical sources in which kaurenoic acid has been identified in the literature. Reported occurrences originate from independent phytochemical investigations employing different extraction, isolation, and analytical methodologies; therefore, the table is intended as a qualitative overview and not a quantitative comparison of kaurenoic acid abundance across species.

Experimental studies show that KA regulates pro-inflammatory mediators, activates apoptosis-related signaling pathways, and inhibits tumor growth markers in preclinical models [1,2,3,4]. These findings have led to increased interest in KA as a potential therapeutic candidate. However, formulation and pharmacokinetic challenges hinder its pharmaceutical development [5,6,7].

**Figure 1:**
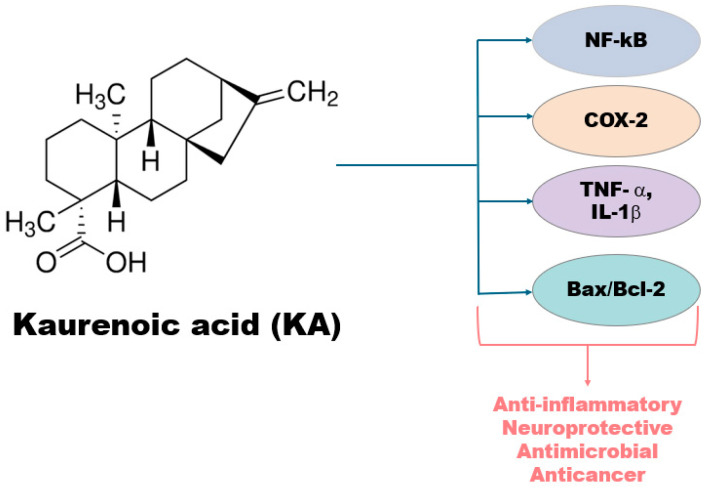
Molecular Structure and Pharmacological Targets of Kaurenoic Acid.

KA exhibits high lipophilicity and poor aqueous solubility, properties that make formulation difficult in conventional dosage forms and may lead to variable systemic exposure in preclinical studies [8]. Figure 1 shows the chemical structure of kaurenoic acid and outlines the postulated and non-clinically validated biological pathways involved in its reported anti-inflammatory, anticancer, and antimicrobial activities.

This figure illustrates the chemical structure of kaurenoic acid (KA), an ent-kaurane diterpenoid characterized by a carboxylic acid functional group and a tetracyclic backbone. Postulated pharmacological targets are highlighted to demonstrate KA’s inhibitory effects on pro-inflammatory pathways (NF-κB, COX-2, TNF-α, IL-1β) and its activation of mitochondrial apoptotic signaling pathways (including modulation of the Bax/Bcl-2 ratio and caspase-3 activation). Arrows depict mechanistic interactions.

Human pharmacokinetics data for kaurenoic acid are unavailable, and systemic exposure reported in animal models varies with formulation and route of administration. Delivering the compound systemically remains a significant challenge for potential clinical development. Lipid-based nanocarrier systems, including liposomes, which have been studied to improve dispersion, stability, increase apparent solubility, and modify the biodistribution of hydrophobic compounds, are considered viable strategies for this compound [9,10,11,12,13].

In this review, we discuss the biopharmaceutics of kaurenoic acid, the limitations affecting its development, and proposed nanocarrier design strategies to overcome these challenges. Available evidence is evaluated to differentiate in vitro preclinical data from in vivo rodent studies and to consider how this information may support future clinical development for potential human use.

**Table 1:** Plant Sources and Experimental Evidence Supporting Pharmacological Activity of Kaurenoic Acid.

Plant Source	Plant Family	Compound Identified	Reported Biological Activity	Experimental Model	References
*Aralia continentalis* Kitag.	Araliaceae	Kaurenoic acid	NF-κB modulation; anti-inflammatory signaling	RAW 264.7 macrophages	[1]
*Copaifera langsdorffii* Desf.	Fabaceae	Kaurenoic acid	Colitis attenuation; cytokine reduction	Rat acetic acid–induced colitis	[4]
*Copaifera* spp. *oleoresin*	Fabaceae	Acid diterpenes, including KA	Pharmacokinetic and metabolic profiling	In vivo rat models; liver microsomes	[5]
*Sphagneticola trilobata* (L.) Pruski	Asteraceae	KA-containing extract	Anti-inflammatory activity	Experimental inflammation models	[3]
Various *Annonaceae* species	Annonaceae	ent-Kaurane diterpenoids	Cytotoxic and anti-inflammatory activity	Phytochemical and preclinical analyses	[14]

To understand the therapeutic potential of kaurenoic acid and the rationale for formulation-based delivery strategies, it is first necessary to examine the pharmacological actions and mechanisms reported in the literature.

## 2. Pharmacological Actions and Therapeutic Potential of KA

Kaurenoic acid (KA) is a biologically active ent-kaurane diterpenoid whose pharmacological effects have primarily been investigated in vitro and in preclinical in vivo models. Under inflammatory conditions, KA has been reported to decrease the expression of pro-inflammatory mediators, including TNF-α, IL-1β, and COX-2, and to disrupt NF-κB signaling pathways [1,2,3]. NF-κB is a key transcriptional regulator of cytokine production and immune activation, and its dysregulation is linked to chronic inflammation and cancer development [15]. The reduction in NF-κB nuclear translocation and subsequent cytokine gene transcription in KA-treated cell models suggests that KA may influence various inflammatory mediators.

Cyclooxygenase-2 (COX-2), a downstream effector of inflammatory signaling, plays a critical role in prostaglandin synthesis and inflammation-associated tumor progression [15]. Reported reductions in COX-2 expression in KA-treated experimental models indicate broader interference with inflammatory signaling pathways, although the magnitude of the effect varies with concentrations and experimental models. In oncology research, KA induces cytotoxic and pro-apoptotic effects in breast, cervical, gastric, and melanoma cell lines [2,4]. Apoptosis induction has been associated with mitochondrial membrane depolarization, activation of caspase-3/9, and changes in the Bax/Bcl-2 ratio. The intrinsic mitochondrial apoptotic pathway is tightly regulated by Bcl-2 family proteins and caspase activation cascades [16,17]. Inflammation-associated tumor progression involves complex crosstalk between cytokine networks and stress-response signaling pathways [15]. While KA-mediated cytokine-linked effects have been reported, these findings apply only to in vitro experimental models, as comparative data across various animal tumor models remain limited. Antimicrobial and metabolic effects have been reported in isolated studies; however, the mechanisms underlying these biological effects are less well characterized than those associated with inflammatory and apoptosis-related pathways. Therefore, experimental evidence supports anti-inflammatory effects and tumor cell apoptosis as principal pharmacological actions of kaurenoic acid with potential therapeutic relevance.

Early pharmacokinetic observations in rodent models indicate limited systemic exposure following oral administration, consistent with KA’s poor aqueous solubility and lipophilicity [8]. These intrinsic physicochemical constraints may limit the attainment of significant therapeutic concentrations in vivo, reinforcing the rationale for formulation-based enhancement strategies. Although these pharmacological observations highlight the biological relevance of kaurenoic acid, translating these effects into therapeutic applications is challenging due to its physicochemical and pharmacokinetic properties.

## 3. Pharmacokinetic and Biopharmaceutical Challenges for Systemic KA Delivery

A detailed examination of the pharmacokinetic and physicochemical determinants underlying these limitations is required (Table 2). The values summarized in this table are derived from independent experimental or computational studies that describe physicochemical parameters relevant to formulation development. Because measurement methods, experimental conditions, and reporting conventions differ across studies, the values should be interpreted as predictive ranges that describe general physicochemical behavior, rather than as standardized values obtained under identical conditions. The intrinsic properties of KA, characterized by high lipophilicity (logP approximately 4–5) and poor aqueous solubility, restrict its suitability for conventional systemic administration. Within the Biopharmaceutics Classification System (BCS), compounds exhibiting low aqueous solubility demonstrate dissolution-limited absorption and formulation-dependent oral bioavailability [18]. Although KA, not yet a drug, has not been formally classified under BCS criteria. However, its reported physicochemical characteristics are consistent with solubility-limited absorption behavior.

Lipophilicity influences membrane partitioning and systemic disposition; however, excessive lipophilicity may reduce aqueous dissolution and luminal availability, limiting effective absorption despite favorable membrane permeability [18]. This balance between solubility and permeability is central to oral drug performance and is particularly relevant for diterpenoid compounds. Preclinical investigations indicate variable oral exposure in rodent models. At the same time, comprehensive pharmacokinetic parameters, such as plasma protein binding, clearance kinetics, and systemic half-life, are not yet characterized in animal species [8]. High plasma protein binding reduces the pharmacologically active unbound fraction and may complicate interpretation of exposure–response relationships [19]. In the absence of defined free-fraction measurements, interpretation of systemic exposure data is difficult. Natural-product-derived diterpenoids exhibit formulation-dependent variability in absorption, rapid metabolic clearance, and limited aqueous dispersibility, all of which may constrain systemic bioavailability [5,6]. These characteristics correspond with the reported behavior of KA in experimental models. Conventional dosage forms, including suspensions and simple oral formulations, have demonstrated limited efficiency in achieving sustained systemic exposure in preclinical settings for compounds with these characteristics. Without solubilization or carrier-based strategies, achieving systemic concentrations is a Herculean task.

Nanocarrier formulations have the potential to modify the apparent pharmacokinetics of poorly soluble compounds, such as kaurenoic acid, by enhancing dispersion, increasing apparent solubility, and altering biodistribution [9,10]. Liposomal and other lipid-based delivery systems are extensively documented in nanomedicine research for their effects on circulation time, clearance pathways, and tissue distribution profiles compared with free compounds [13,20]. These attributes support further investigation of lipid-based nanocarrier systems as delivery vehicles for KA, although comprehensive pharmacokinetic studies are yet to be conducted to accurately characterize systemic exposure.

**Table 2:** Physicochemical and Pharmacokinetic Determinants Influencing Systemic Delivery of Kaurenoic Acid.

Parameter	Evidence-Based Description	Biopharmaceutics Relevance	References
Molecular Weight	302.45 g/mol	Compatible with passive diffusion	[5]
LogP	~4–5 (literature-reported range)	High lipophilicity; favors lipid-phase partitioning	[5]
Aqueous Solubility	Practically insoluble in water	Requires a lipid-based or solubilization strategy	[5]
Oral Exposure	Low systemic exposure in rodent models; variability reported	Oral bioavailability is formulation-dependent	[5]
Plasma Protein Binding (PPB)	High binding tendency suggested in PK profiling studies	High PPB may reduce the free fraction	[5,21]
Clearance/Half-Life	Rapid systemic clearance reported in rodents	Sustained exposure unlikely without carrier-based effect	[5]
Key Biopharmaceutical Limitation	Poor solubility; high lipophilicity; exposure variability	Strong rationale for nanocarrier-based development	[5,22]

The intrinsic physicochemical limitations discussed above are the main reasons for exploring carrier-based delivery systems to enhance the dispersion, stability, and systemic bioavailability of kaurenoic acid.

## 4. Nanoliposomal Delivery Systems

**Figure 2:**
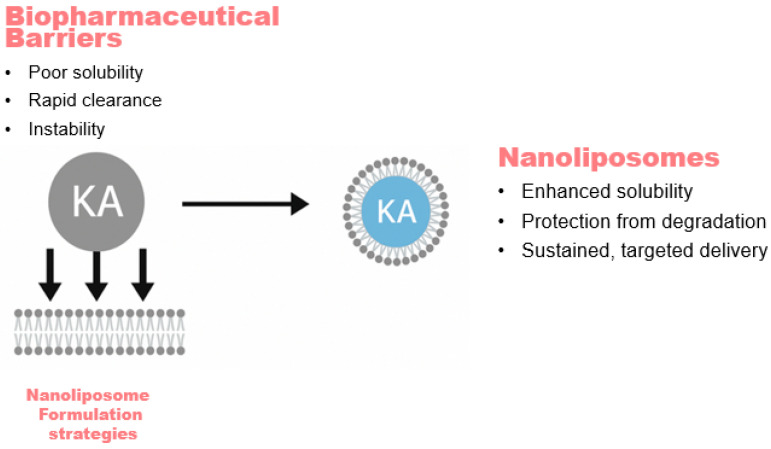
Schematic Representation of Biopharmaceutical Barriers and Nanoliposomal Encapsulation Strategies.

Nanocarrier-based delivery strategies have been extensively explored to address the biopharmaceutical limitations associated with hydrophobic compounds such as kaurenoic acid. Nanoliposomes, vesicular carriers typically <100 nm in diameter composed of phospholipid bilayers, can efficiently incorporate hydrophobic molecules within their lipid membranes. This structural arrangement enhances the apparent solubility of poorly water-soluble compounds, protects the encapsulated payload from chemical and enzymatic degradation, and can prolong systemic circulation time by reducing rapid clearance (see Figure 2). The nanoscale size and biomimetic lipid composition of nanoliposomes further support improved stability and favorable pharmacokinetic behavior, making them a widely investigated delivery system for hydrophobic compounds.

As illustrated in Figure 2, nanoliposomes can shield KA from enzymatic metabolism and photodegradation, significantly extending its shelf life and in vivo stability. Among lipid-based nanocarriers, nanoliposomes are widely used for the delivery of hydrophobic compounds. Their phospholipid bilayer architecture enables the partitioning of lipophilic molecules such as KA into the hydrophobic membrane domain, thereby improving their apparent solubility and dispersion stability in aqueous environments [9,10]. Liposomal formulations have received regulatory approval for multiple oncologic and anti-inflammatory indications, demonstrating the established role of liposome-based delivery systems in modern drug development [13,19]. Encapsulation within nanoscale vesicles may influence biodistribution through passive accumulation mechanisms historically associated with the enhanced permeability and retention (EPR) effect [20]. However, the magnitude and reproducibility of EPR-mediated tumor accumulation vary widely between tumor types and species, reflecting pronounced intertumoral heterogeneity in vascular permeability and stromal structure [17]. Consequently, the contribution of passive targeting should be interpreted with caution when extrapolating preclinical observations to animal and human models.

Surface modification strategies such as PEGylation are commonly used to reduce opsonization and prolong systemic circulation time. PEGylated liposomes result in extended plasma half-life and reduced uptake by the mononuclear phagocyte system in multiple nanomedicine studies [13,19]. Nevertheless, repeated administration of PEGylated formulations has been associated with accelerated blood clearance (ABC) phenomena and anti-PEG antibody formation in some experimental models, raising immunogenicity considerations during clinical development [22].

In addition to surface modification, the method used to prepare liposomes can substantially influence their physicochemical characteristics, which determine their in vivo behavior. Preparation techniques, including thin-film hydration, reverse-phase evaporation, ethanol injection, and microfluidic mixing, yield liposomes with varying size distributions, lamellarity, and encapsulation efficiencies [11,12]. Because these attributes influence loading, release kinetics, and systemic disposition, the fabrication method is an important formulation variable to consider in preclinical studies. Microfluidic preparation methods improve reproducibility and scalability compared with conventional batch techniques, although formulation-specific optimization remains necessary. In experimental models, liposomal KA formulations alter biodistribution patterns and produce enhanced pharmacodynamic responses compared with free KA in selected tumor models [2,4]. These observations appear to depend strongly on formulation composition and experimental model. Key liposomal and nanocarrier strategies relevant to KA delivery are summarized in Table 3. The formulation strategies summarized are from separate studies employing heterogeneous formulation techniques, carrier compositions, and characterization methods. Reported parameters are presented to illustrate general formulation strategies explored for kaurenoic acid delivery and should not be interpreted as direct head-to-head comparisons between nanocarrier systems.

**Table 3:** Nanocarrier Platforms Investigated for Delivery of Hydrophobic Compounds Including Kaurenoic Acid.

Delivery System	Structural Basis	Encapsulation Strategy for KA	Advantages	Limitations	References
Conventional Liposomes	Phospholipid bilayer vesicles	Bilayer partitioning accommodates lipophilic KA	Established platform; clinical precedent	Aggregation, leakage	[10,11,12,13]
PEGylated Liposomes	PEG surface-modified vesicles	Prolonged systemic circulation	Reduced opsonization	Accelerated blood clearance risk	[21,23]
Microfluidic Liposomes	Continuous-flow vesicle fabrication	Controlled particle size distribution	Scalable manufacturing	Equipment requirements	[13]
Polymeric Nanoparticles	Biodegradable polymer matrices	Hydrophobic compound encapsulation	Controlled release	Polymer toxicity concerns	[19]
Theranostic Liposomes	Co-loaded therapeutic + imaging systems	Conceptually compatible with KA	Imaging-guided monitoring	Increased regulatory complexity	[20]

Understanding the biological performance of nanocarrier systems requires careful consideration of the physicochemical properties that control carrier stability, loading, and release behavior.

## 5. Physicochemical Characterization and Biopharmaceutical Performance of KA Nanocarriers

Physicochemical characterization is essential for interpreting the biological performance of liposomal and nanocarrier formulations developed for hydrophobic compounds such as kaurenoic acid (KA). Key parameters typically evaluated include particle size distribution, polydispersity index (PDI), zeta potential, encapsulation efficiency, and drug release kinetics. These nanoformulation attributes influence formulation stability, biodistribution, and pharmacokinetics [23].

Among these parameters, particle size plays a central role in systemic distribution and clearance. For liposomal formulations intended for KA delivery, nanocarriers in the 50–200 nm range are generally considered optimal for prolonged circulation and potential tumor accumulation. In contrast, larger vesicles may be rapidly taken up by cells of the mononuclear phagocyte system [13]. Control of size distribution is therefore important during formulation development. Narrow size distributions, reflected by low PDI values, improve reproducibility and reduce batch-to-batch variability in nanoformulation preparation [23]. Surface charge characteristics also contribute to nanocarrier stability and biological interaction. Zeta potential is widely used to assess the colloidal and physical stability of nanocarrier formulations by quantifying the electrostatic interactions that govern particle aggregation. Highly charged particles tend to exhibit stronger electrostatic repulsion, which can reduce aggregation during storage and systemic circulation. However, extreme surface charges may also influence protein adsorption and immune recognition, potentially altering the in vivo pharmacokinetic behavior of liposomal KA formulations [24].

Encapsulation efficiency is another critical parameter, particularly for hydrophobic compounds such as KA. Incorporation of lipophilic molecules within the lipid bilayer can substantially improve apparent aqueous dispersibility. Efficient encapsulation also minimizes the presence of free KA fractions that may contribute to precipitation or rapid systemic clearance [9,10]. Drug release kinetics further influence therapeutic performance. Sustained-release profiles may prolong exposure to target tissues while reducing peak systemic concentrations, thereby contributing to toxicity. Conversely, excessively stable formulations may limit effective bioavailability if KA release becomes insufficient [23]. Reliable measurement of these physicochemical properties requires complementary analytical techniques. Liposomal characterization often involves dynamic light scattering (DLS) to determine particle size, transmission electron microscopy (TEM) to assess morphology, and chromatographic methods to quantify encapsulated and free fractions [25]. These analytical methods define the key physicochemical attributes required to interpret pharmacokinetic and pharmacodynamic outcomes for KA-containing nanocarrier formulations. A summary of physicochemical parameters reported for KA nanocarrier formulations is presented in Table 4. This table summarizes key physicochemical attributes commonly evaluated during characterization of liposomal formulations and the analytical methods used to assess them. The parameters are compiled from independent studies and presented to illustrate general characterization considerations for kaurenoic acid nanoformulations.

Once these physicochemical attributes are determined, their effects on biological performance can be evaluated through preclinical pharmacological and pharmacokinetic studies.

## 6. Preclinical Evaluation of Nanocarrier-Based KA Systems

Experimental studies in inflammatory and oncologic models indicate that encapsulated KA exhibits altered pharmacodynamic responses, likely reflecting improved formulation stability and modified biodistribution profiles associated with nanoparticle delivery [2,4]. Several investigations have reported enhanced antitumor activity in murine models following administration of liposomal KA formulations compared with non-encapsulated KA [2]. Reported findings include reductions in tumor volume, changes in inflammatory mediator expression in the tumor microenvironment, and increased expression of apoptotic markers in tumor tissues [2]. However, variability in tumor models, dosing regimens, and formulation preparation methods limits direct comparison with other studies.

Similar observations have been reported in inflammatory disease models. Liposomal and other lipid-based KA formulations have reduced inflammatory cytokine levels and improved histopathological outcomes in experimental colitis and related inflammation-associated conditions [4]. These findings are consistent with the anti-inflammatory signaling pathways described in earlier mechanistic studies. From a therapeutics-delivery perspective, interpretation of these biological effects requires consideration of formulation-dependent pharmacokinetics. Pharmacokinetic investigations of KA-containing lipid formulations indicate that encapsulation can alter circulation time and tissue distribution compared with free KA [5]. Lipid-based carriers can reduce rapid systemic clearance and improve the dispersion of hydrophobic molecules in biological fluids. However, information regarding quantitative pharmacokinetic parameters, including absolute bioavailability, systemic clearance, and exposure–response relationships, is scarce. Interpretation of nanocarrier performance must also consider formulation variables that influence in vivo behavior. Lipid composition, particle size distribution, surface charge, and encapsulation efficiency are established determinants of nanocarrier biodistribution and systemic exposure. Variations in these attributes can influence observed therapeutic responses and complicate comparisons among liposomal KA formulations prepared using different fabrication techniques [23,24,25].

Available preclinical evidence indicates that nanocarrier-mediated delivery can modify the pharmacological behavior of KA. However, rigorous comparison of KA delivery approaches requires well-controlled formulation studies and quantitative pharmacokinetic analysis to distinguish formulation-driven effects from variability related to experimental models. Representative preclinical investigations of free and nanocarrier-based KA formulations are summarized in Table 5, which outlines experimental models, dosing conditions, pharmacodynamic outcomes, and available pharmacokinetic observations. The experimental models, dosing conditions, and results summarized in this table were compiled from independent in vitro and in vivo investigations using heterogeneous experimental designs. The table provides a qualitative overview of reported pharmacological observations and should not be interpreted as a direct comparison of efficacy between different studies.

Although these experimental findings are promising, advancing nanocarrier-enabled kaurenoic acid formulations to clinical testing requires careful consideration of regulatory and pharmaceutical development requirements.

## 7. Regulatory and Pharmaceutical Development Considerations for KA Nanocarrier Systems

Despite encouraging experimental findings, advancement of kaurenoic acid (KA) nanocarrier systems toward clinical development requires meeting established regulatory standards for pharmaceutical quality, safety, and manufacturing control. For lipophilic compounds such as KA, formulation design and regulatory evaluation are closely linked because physicochemical properties, including high lipophilicity and poor aqueous solubility, directly influence systemic exposure and therapeutic performance. Regulatory agencies such as the U.S. Food and Drug Administration (FDA) and the European Medicines Agency (EMA) require detailed physicochemical characterization when evaluating lipid-based nanomedicines. For liposomal KA formulations, this characterization typically includes particle size distribution, polydispersity index, surface charge (zeta potential), encapsulation efficiency, and formulation stability [21]. These attributes are recognized as critical quality attributes (CQAs) for nanocarrier systems because small variations in particle properties can significantly influence biodistribution, systemic exposure, and pharmacological activity.

Manufacturing reproducibility is a crucial requirement in pharmaceutical development. Nanoscale formulations exhibit heightened sensitivity to formulation conditions, whereby even minor variations in lipid composition, mixing parameters, or solvent removal processes can result in significant alterations in particle size, lamellarity, and drug loading. Application of Quality-by-Design (QbD) principles is therefore increasingly recommended for lipid-based nanocarrier development [27]. QbD approaches facilitate identification of critical material attributes and process parameters that control formulation characteristics such as particle size distribution, encapsulation efficiency, and release behavior in liposomal KA formulations. Implementation of these formulation strategies can improve batch-to-batch consistency and support scale-up during later stages of pharmaceutical development.

Beyond formulation quality considerations, regulatory evaluation also requires a comprehensive pharmacokinetic characterization. Given the limited pharmacokinetic information currently available for KA, development of KA nanocarrier formulations should incorporate quantitative evaluation of systemic exposure, tissue distribution, and clearance relative to the free compound. These investigations typically involve analysis of plasma concentration–time profiles, tissue biodistribution patterns, and metabolic stability using validated analytical methods [20]. Demonstration that nanocarrier encapsulation meaningfully alters KA pharmacokinetics, such as by prolonging circulation time or altering tissue distribution, is necessary to justify further clinical development.

Safety evaluation is an additional critical component of nanocarrier development evaluation. Liposomal and lipid-based systems can interact with circulating proteins and immune components, influencing clearance pathways and potentially eliciting immunological responses. Complement activation-related pseudoallergy (CARPA) has been reported with certain lipid nanoparticles and must therefore be considered in preclinical safety screening [28]. These safety evaluations generally include immunotoxicity testing, repeated-dose toxicity studies, and assessment of complement activation in relevant animal models.

Finally, the natural-product origin of kaurenoic acid introduces additional regulatory considerations related to chemical identity and manufacturing control. Development of KA-based formulations requires characterization of compound purity, structural identity, and impurity profiles to ensure reproducibility of the active pharmaceutical ingredient. Variability in phytochemical composition may arise if botanical source materials are not carefully standardized. Accordingly, isolation procedures, validated analytical characterization, and impurity profiling must be clearly defined in the chemistry, manufacturing, and controls (CMC) documentation before KA formulations can progress to clinical evaluation. Considering these regulatory considerations, several key research questions persist that will influence the feasibility of advancing kaurenoic acid nanocarrier systems toward clinical trials.

## 8. Perspectives on Pharmaceutical Development Considerations for Kaurenoic Acid Nanocarriers

Although KA shows promising pharmacological activity in experimental models, several critical questions must be addressed before clinical development can be realistically considered. First, a comprehensive pharmacokinetic characterization of KA is required to define systemic exposure parameters, including absorption, distribution, metabolism, and elimination profiles. Such information is essential for establishing dose–response relationships and for interpreting pharmacodynamic outcomes. Second, standardized comparative studies evaluating free KA versus nanocarrier-based systems are required. Many existing investigations employ different tumor models, dosing regimens, and formulation strategies, making direct comparison difficult. Controlled studies using comparable experimental designs would provide stronger evidence regarding the contribution of nanocarriers to therapeutic performance. Third, a comprehensive toxicological assessment is essential. Although KA exhibits low toxicity in several preclinical models, a comprehensive toxicological evaluation, including repeated-dose screening, tissue distribution analysis, and immunological response profiling, has not been conducted. These studies are vital for assessing whether KA-based formulations can safely advance human trials. Finally, nanocarrier engineering strategies, such as ligand-modified liposomes, stimuli-responsive carriers, and hybrid lipid–polymer nanoparticles, are being investigated to improve the targeted delivery of hydrophobic therapeutic agents, including compounds with physicochemical properties similar to KA [33,34,35,36]. These nanocarrier strategies may improve delivery specificity and enhance biopharmaceutics and pharmacokinetics parameters. Addressing these formulation, pharmacological, and regulatory challenges will be essential for determining the long-term therapeutic potential of kaurenoic acid-based nanocarrier systems.

## 9. Conclusions

The available literature indicates that the therapeutic potential of kaurenoic acid is closely linked to the development of formulation strategies to overcome its intrinsic biopharmaceutical limitations. Kaurenoic acid is a biologically active diterpenoid with anti-inflammatory and cytotoxic properties in in vitro experimental models. Studies have shown effects on inflammatory signaling pathways, induction of apoptosis, and reduced expression of pro-inflammatory mediators. However, intrinsic physicochemical properties, including poor aqueous solubility and high lipophilicity, limit its systemic exposure and complicate conventional formulation strategies. Lipid-based nanocarriers, particularly liposomes, are a promising approach for enhancing the delivery of hydrophobic molecules such as KA. Encapsulation strategies can enhance stability, alter biodistribution patterns, and potentially improve pharmacodynamic responses in preclinical models. Nevertheless, reported benefits depend on formulation characteristics and pharmacokinetic and pharmacodynamic studies for confirmation. Future development efforts should focus on physicochemical characterization, reproducible nanocarrier formulations, safety, efficacy, and pharmacokinetic studies. Conducting these studies with quality-by-design principles will be essential for advancing nanocarrier-enabled KA formulations toward clinical development.

## Figures and Tables

**Table 4:** Critical Quality Attributes and Characterization Parameters Relevant to Liposomal Delivery of Kaurenoic Acid.

Characterization Parameter	Analytical Method	Relevance to KA Delivery Performance	References
Particle Size	Dynamic light scattering (DLS)	Influences systemic distribution, clearance, and potential tumor accumulation of liposomal KA	[11,13]
Polydispersity Index	DLS-derived metric	Indicator of size distribution uniformity and formulation reproducibility	[11]
Zeta Potential	Electrophoretic mobility measurement	Indicator of colloidal stability and physical stability of KA nanocarrier dispersions	[13]
Morphology	Transmission electron microscopy	Confirms vesicular structure and structural integrity of liposomal KA formulations	[26]
Encapsulation Efficiency	Chromatographic quantification	Determines the proportion of KA incorporated within the lipid bilayer	[13]
In vitro Release	Dialysis or diffusion testing	Characterizes the release kinetics of KA from liposomal carriers	[25]
Stability Testing	Accelerated and real-time studies	Evaluates the physical and chemical stability of KA liposomal formulations during storage	[27,28,29]

**Table 5:** Representative Preclinical Studies Evaluating Free and Nanocarrier-Based Kaurenoic Acid (KA).

Model/Indication	KA Formulation	Dose and Route	Reported Biological Outcomes	Pharmacokinetic/Exposure Information	Safety Observations	References
Neuropathic Pain (CCI model, murine)	Free KA	1–10 mg/kg, intraperitoneal	Reduced hyperalgesia; decreased TNF-α and IL-1β expression	Plasma concentrations not reported	No overt acute systemic toxicity	[2]
Experimental Colitis (rat)	Free KA	10–50 mg/kg, oral	Reduced inflammatory markers; improved histopathological scores	Limited pharmacokinetic characterization	Short-term evaluation only	[4]
Melanoma Xenograft (murine)	Free KA	5–20 mg/kg, intraperitoneal	Tumor growth inhibition	No formal pharmacokinetic profiling	Dose-dependent toxicity at higher exposure	[30]
Breast Cancer (in vitro)	KA nanoformulation	1–50 µM	Reduced cell migration and increased apoptotic signaling	Not applicable (cell culture model)	Cytocompatible within tested range	[31]
Cervical Cancer (in vitro)	Free and nano KA	5–40 µM	Mitochondrial depolarization; Bax/Bcl-2 expression changes	Not applicable (cell culture model)	Assay-dependent interpretation	[6]
Murine Tumor Model	Liposomal KA	5–15 mg/kg, intravenous	Greater tumor growth inhibition compared with free KA	Altered biodistribution patterns reported	Short-term tolerability acceptable	[10,11,12,13,14,15,16,17,18]
Genotoxicity Assessment	Free KA	Ames test conditions	No mutagenicity detected	Not applicable	Long-term carcinogenic risk not established	[32]

## Data Availability

No new data were created or analyzed in this study. Data sharing is not applicable to this article.

## Abbreviations

The following abbreviations are used in this manuscript:

Abbreviation	Definition
ADME	Absorption, distribution, metabolism, and excretion
CMC	Chemistry, manufacturing, and controls
EMA	European Medicines Agency
EPR	Enhanced permeability and retention
FDA	U.S. Food and Drug Administration
GMP	Good manufacturing practice
IND	Investigational new drug
ISO	International Organization for Standardization
KA	Kaurenoic acid
LC–MS/MS	Liquid chromatography–tandem mass spectrometry
NLC	Nanostructured lipid carrier
PEG	Poly(ethylene glycol)
PK	Pharmacokinetics
QA	Quality assurance
QbD	Quality by design
RAW 264.7	Murine macrophage cell line
SLN	Solid lipid nanoparticle
WHO	World Health Organization
